# A novel osteogenesis technique: The expansible guided bone regeneration

**DOI:** 10.1177/2041731412441194

**Published:** 2012-04-04

**Authors:** Osama Zakaria, Marwa Madi, Shohei Kasugai

**Affiliations:** Department of Oral Implantology and Regenerative Dental Medicine, Tokyo Medical and Dental University, Tokyo, Japan

**Keywords:** tissue engineering, facilitated endogenous repair, in vivo bone incubator, in situ bone incubator, expansible guided bone regeneration, supraosteal bone neogenesis, de novo bone, bone induction

## Abstract

Guided bone regeneration is a unique osteogenesis technique that requires a barrier membrane under periosteum to create space for bone regeneration. However, creating sizeable spaces is clinically not commonly feasible. A titanium plate and a thin silicone membrane were surgically layered on each calvaria of eight rabbits. Then, the periphery of the silicone membrane was fixed by a plastic ring to the underlying bone using titanium micro screws. After 1 week, a 5-mm-length titanium screw was used to elevate the titanium plate, which in turn elevated the silicone membrane together with overlying soft tissue in a rate of 1 mm/day for 5 days to create a secluded space. Animals were killed at 2 months (n = 4, group 1) and 4 months (n = 4, group 2) after the elevation. Histological and microradiographical analyses demonstrated creation of an amount of de novo bone formation (68.2 ± 22 mm^3^ in group 1 and 70.3 ± 14 mm^3^ in group 2) in the sizeable created spaces (207.1 ± 31 mm^3^ in group 1 and 202 ± 21 mm^3^ in group 2) without exposure of the device. This novel osteogenesis technique, “expansible guided bone regeneration,” created a substantial in vivo incubator without applying growth factors or osteoprogenitor cells. Creating a growing space over the secluded surface allowed the development of normal biological healing process occurring on the bone surface into a regenerative process, generating bone outside the genetically determined skeletal bone. This technique is a new tissue engineering approach stimulating endogenous tissue repair without applying cells or factors exogenously.

## Introduction

Bone augmentation is clinically required in orthopedic and dental fields, and autogenous bone graft is still a gold standard for bone augmentation. However, limitation of the harvestable bone volume and inflammation of the donor site are problems. Effective and less invasive alternative for bone augmentation has been absolutely required. “Cells,” “signal molecules,” and “scaffold” are three key players prerequisite for tissue regeneration including bone regeneration. The current tissue engineering strategy is exogenously applying one or combination of these three key players to the regenerative side. Indeed, it has been reported that application of bone-inducing signal molecules, such as bone morphogenetic protein (BMP) 2 or 7, is effective in bone augmentation.^1^ Furthermore, effectiveness of applying osteogenic cells from bone marrow or periosteum in bone regeneration has also been reported.^2^ Numerous preclinical studies and some clinical successes indicated that these tissue engineering approaches are scientifically practical.^3–5^ However, restricted by high cost and complexity of production, the tissue engineering products have not yet been commercially effective.^6^ These issues may retain tissue engineering as a costly and exclusive treatment for those who can afford.^7^ Some studies pointed out the negative effect of labor-intensive production and distribution methods of engineered tissues.^8^

Other alternatives to these tissue engineering strategies have been explored to regenerate bone. Simon et al.^9^ used an “in situ incubator” technique to avoid the need for expansion of periosteal cells in tissue culture. They evaluated the response of mechanical release of periosteum to stimulate the underlying cambium cells. Procedures involved sharply incising through the superficial periosteal fibrous layer down to and scoring the cortical bone surface. This technique resulted in increasing the number of cambium cells and bone generation in situ under the proliferated cells. They concluded that this technique can be cost-effective for periosteum transplantation.^9^ In 2005, Stevens et al.^10^ showed that large volumes of bone can be produced in a predictive manner without exogenously applying the three key players, if the space is provided by injecting biocompatible gel under periosteum. More recently, we and others have been reported that gradual periosteum elevation creating a space over bone surface results in new bone formation in this space.^11–16^ These studies clearly suggest that elevating periosteum and providing space over bone surface elicit new bone formation. However, the invasion of the created space with highly competitive nonosteogenic soft tissue and poor quality of the newly formed bone are the main drawbacks of this technique.^17,18^ In addition, periosteum viability may be commonly reduced probably due to age or disease.^9,10^

On the other hand, de novo supra osseous bone formation using guided bone regeneration (GBR) is a unique clinical technique for bone augmentation in which a barrier membrane is placed under periosteum providing a space over bone surface resulting in new bone formation in this space.^19–23^ It is clear that in GBR, migration of soft tissue including periosteum into the space under the barrier membrane is eliminated providing a favorable space for bone regeneration. However, creating a sizeable GBR space is not commonly feasible clinically.^24^ The technique requires excellent soft tissue management to avoid the major complication of premature membrane exposure and the subsequent bacterial contamination. This is generally due to insufficient soft tissue coverage leading to excessive tension of tissues during flab closure.^25,26^

Indeed, in 2007, Evans et al.^7^ have proposed the new concept for tissue regeneration: ‘‘facilitated endogenous repair” as an alternative biologically based approach that simplifies the technology and stimulates natural healing in situ. It depends on harnessing the intrinsic regenerative potential of endogenous tissues avoiding the ex vivo culture of autologous cells and the requirement for synthesized scaffolds. This approach can reduce associated clinical maneuvers. It is interesting to note that periosteum elevation and GBR are also utilizing “endogenous regenerative potential.”

In our previous “periosteum elevation” study, it was very likely that new bone was mainly produced from the basal bone, not from the periosteum.^11^ Thus, it is reasonable for us to speculate that gradual elevation of the barrier membrane, which is initially placed on the bone surface, gradually increasing the space over the bone surface could produce new bone efficiently. Very recently, it has been reported that elevation of periosteum with collagen membrane covering the perforated titanium plate produces more new bone compared to the elevation with the perforated titanium plate, which strongly support our speculation. However, in that study, the space created on the bone surface was not sealed, permitting the surrounding connective tissue invasion to this space.^27^

The goal of this study was to determine the validity of our model as an in situ bioreactor, which is designed to induce supra osseous de novo bone. Producing this bioreactor is based on gradual creation of an artificial space between the calvarial bone surface and the overlying soft tissue using a novel osteogenesis technique “the expansible guided bone regeneration (EGBR)”. This technique depends on the combined use of two techniques: periosteal distraction and guided bone regeneration. In generating this model, we hypothesized the following:

1. Gradual creation of sizeable space between periosteum and an activated surface would boost normal healing process taking place typically on the bone surface in the first week after activation into a regenerative action that results eventually into reconstitution of the space with living functional bone.
2. Soft tissue will continue to be gradually expanded and will continue to be intact and not expose the underlying GBR membrane until the end of experiment.

## Materials and methods

Eight Japanese male white rabbits, weighing from 2.5 to 3 kg, were used. The experimental protocol was approved by the Committee of Animal Experiments in Tokyo Medical and Dental University (Approval No. 01202241A).

### Construction of the device

**Figure 1.**
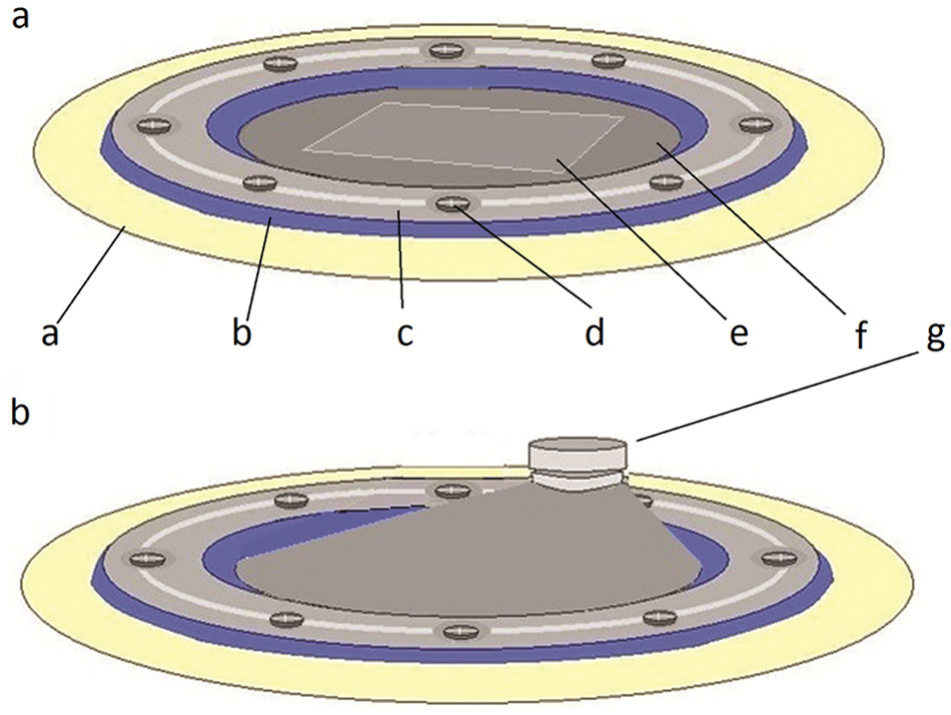
An illustration showing the device: (a) before and (b) after activation. a: calvarial bone; b: silicone ring; c: plastic ring; d: fixation screw; e: elevation plate; f: silicone membrane; g: elevation screw

The EGBR device has the following six components (Figure 1(a) and (b)):

1. Rectangular shape titanium elevating plate (16 mm × 10 mm × 0.5 mm), in which three holes were prepared: two holes for fixation screws and one serrated hole for activation screw
2. Titanium fixation mini screws (3 mm in length and 1 mm in diameter)
3. Titanium elevating screw (5 mm in length and 2 mm in diameter)
4. Plastic ring (24 mm × 18 mm × 0.5 mm external dimensions) with eight equidistant fixation holes
5. Silicone membrane (0.05 mm thick) contains one hole for activation screw (1.8 mm wide)
6. Silicone ring (0.2 mm thick) has the same dimensions of the plastic ring and is located directly beneath it

### Surgery

Preoperatively intramuscular ketamine (50 mg/kg Ketalar; Sankyo, Tokyo, Japan) and thiopental sodium (25 mg/kg Ravonal; Tanabe, Tokyo, Japan) were injected to anesthetize animals. In addition, the surgical site was injected with 1.8 mL of a local anesthetic (2% xylocaine/epinephrine 1:80,000; Dentsply Sankin, Tokyo, Japan) before surgery.

**Figure 2.**
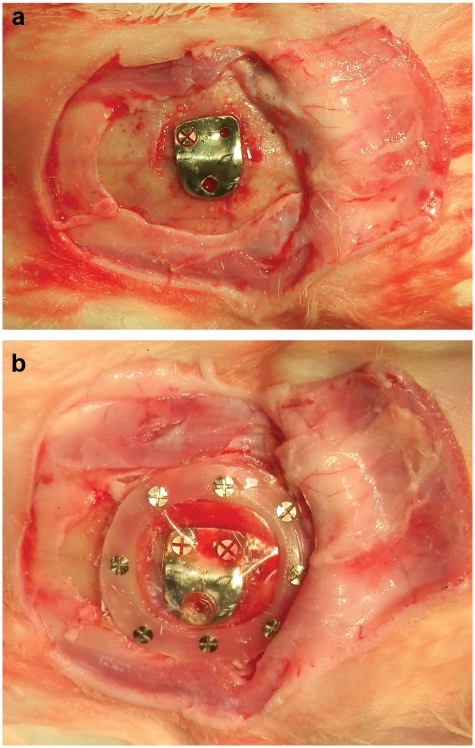
Pictures of the device in operative site: (a) titanium plate fixed to the calvarial bone by fixation screw and (b) whole device installed.

All operations were performed under aseptic conditions. During the surgery, vital signs were closely monitored. Before surgery, the foreheads of the animals were shaved and disinfected with tincture of 1% iodine solution. The calvarial bone was exposed by a U-shaped skin and subperiosteal incision. The skin flap was reflected followed by the periosteum, which was retracted and kept away from the operative site. Under irrigation with saline, surface activation was made with a round bur of number 4 in the external cortical plate of the occipital bone. The elevating plate of the device was first placed over the activated area and then two mini screws were used to fix the plate to bone surface from one of its ends (Figure 2(a)). Then, the plastic ring to which the silicone membrane is secured was placed to cover the elevating plate and then fixed to the calvarial bone by eight micro screws (Figure 2(b)). The flab was then sutured back in layers. No antibiotic was given to the animals.

One week later, a soft tissue incision over the screw place of the elevating plate was incised. The elevating screw was advanced in the screw hole to attach to the elevating plate. The screw passed through the soft tissue and the underlying silicone membrane through a circular hole in the membrane (which is less than the diameter of the screw by 0.2 mm).The end of the screw was flat and was resting on the external cortical layer of the calvarial bone. Rotating the screw 360° causes one side of the titanium elevating plate to be raised by 1 mm and consequently move the overlying silicone membrane and soft tissue up. Activation rate of 1 mm/day was applied for 5 days to the plate causes the overlying silicone membrane to expand gradually and finally take a shape of a tent (Figure 2(a) and (b)).

The animals were kept in a standard cage in an experimental animal room and fed a standard laboratory diet and water during the observation period. Group 1 animals (n = 4) were killed after a consolidation period of 2 months followed by group 2 (n = 4) at 4 months with a lethal dose of thiopental sodium. The entire cranial bone was removed and fixed for 14 days in neutral 10% formalin.

### Micro-computed tomography analysis

After fixation specimens were scanned using a high-resolution micro-computed tomography (CT) imaging system (SMX-90CT; Shimadzu, Kyoto, Japan) continuously in increments of 60 µm. The bone images were extracted by processing the gray scale images using a median filter to remove noise and a fixed threshold to extract the mineralized bone phase. Following phantom calibration of the images, scanned images were analyzed with three-dimensional (3D) image analysis software (TRI/3D-BON; Rotac system engineering, Tokyo, Japan) from which volume of newly created space (Vs) and new bone tissue volume (Bv) were obtained.

**Figure 3.**
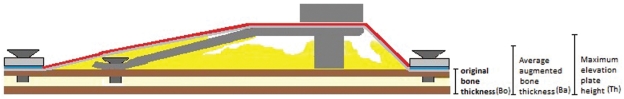
Illustration showing a cross-sectional view of the installed device.

From each scanned specimen, 10 serial sagittal images were obtained (1 image/mm).^28^ Images were automatically corrected for brightness and contrast and then were converted into 8-bit gray scale before measurement. Then images were used serially to measure the average height of the original bone (Bo), average height of augmented bone (Ba), and maximum height of titanium plate (Th)^28^ (Figure 3) with image analysis software (Image j, 1.43 Hz; NIH, Bethesda, MD, USA). Average and standard deviation were obtained from calculations of each segment.

### Statistics

Statistical analysis was performed with SPSS statistical package. Descriptive statistics included mean and standard deviation of all the variables were calculated. Mann–Whitney U test was used to compare the amount of the newly formed bone volume (Bv) and average height of augmented bone (Ba) between the two groups. The level of significance was set to 95%.

### Histological processing

Following fixation, calvarial bone was dehydrated in ascending grades of ethanol and then embedded in polyester resin (Technovit 7200; Heraeus Kulzer GmbH, Wehrheim, Germany). The distraction devices were kept in place, and then sections were cut (Exakt, Mesmer, Ost Einbeck, Germany) and ground to a thickness of about 100 µm. The sections were finally stained with 0.1% toluidine. Histological examinations were performed in a BZ-8000 microscope (Keyence, Osaka, Japan), and the data were analyzed using BZ-Analyzer software (Keyence).

## Results

In all animals, normal dietary habit was resumed immediately after cessation of general anesthesia effect. An infection symptom was detected in one animal after 1 week of device insertion and was excluded from the experiment. No inflammation was observed in all other animals. All devices remained rigidly fixed to the calvarium during the experiment. They were totally concealed under the soft tissue and the membrane did not show exposure during activation and until the time of kill. Activation screws were easily adjusted and remained attached to the device until the end of the experiment. In some animals (two animals in group 1 and one animal in group 2), while elevation of the plate, the screw hole in the membrane was dilated for few millimeters giving the chance for soft tissue to locally grow in the adjacent part in the space. However, the membrane was intact in all other areas protecting adequately the new bone. This problem was overcome later by placing the part of the membrane anterior to the screw in a folded state. In some animals, the 2-mm soft tissue incision could have closed before complete activation of screw. This imposed activation in a higher pace than intended in some animals.

### Micro-CT

**Figure 4.**
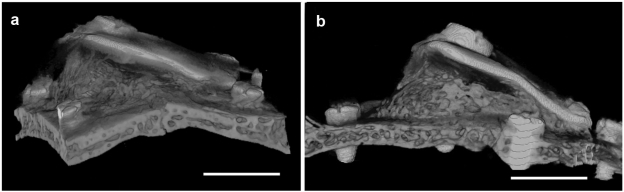
Lateral view shows the image of inclined titanium plate over the original bone. The space created in between them was almost occupied by newly formed bone, which extends outside the titanium plate boundary: (a) group 1 and (b) group 2. scale bar=4.5mm

**Figure 5.**
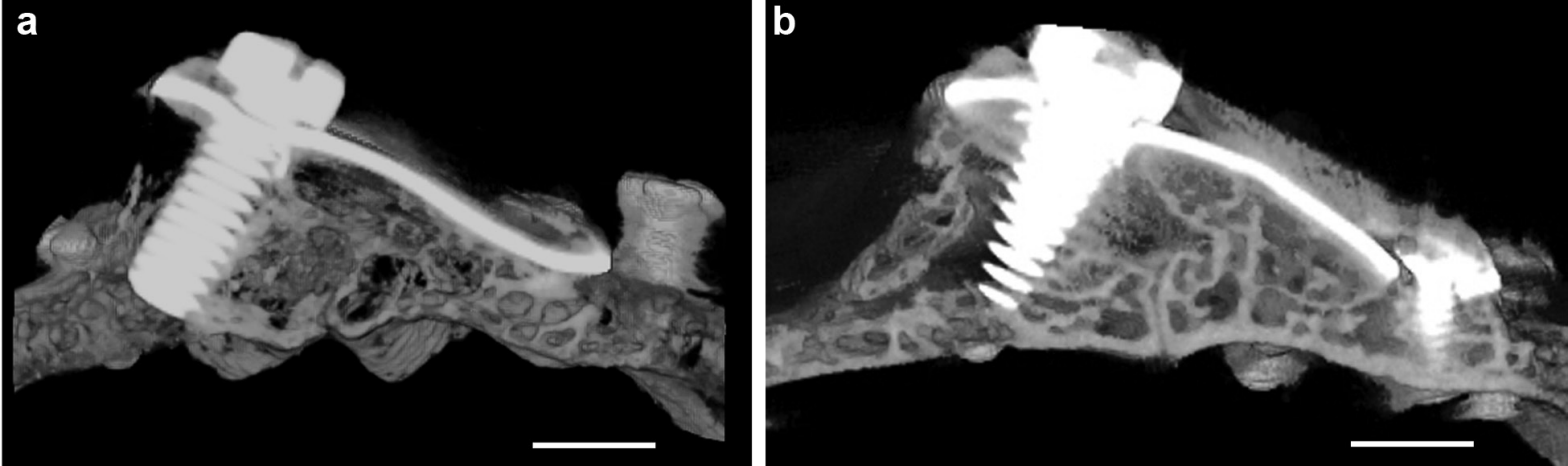
Longitudinal section view shows a view of a triangle whose sides are formed by original bone, titanium plate, and newly formed bone. Note that the thickness of new bone (b) is almost equivalent to the original one: (a) group 1 and (b) group 2. scale bar =2.5mm

**Figure 6.**
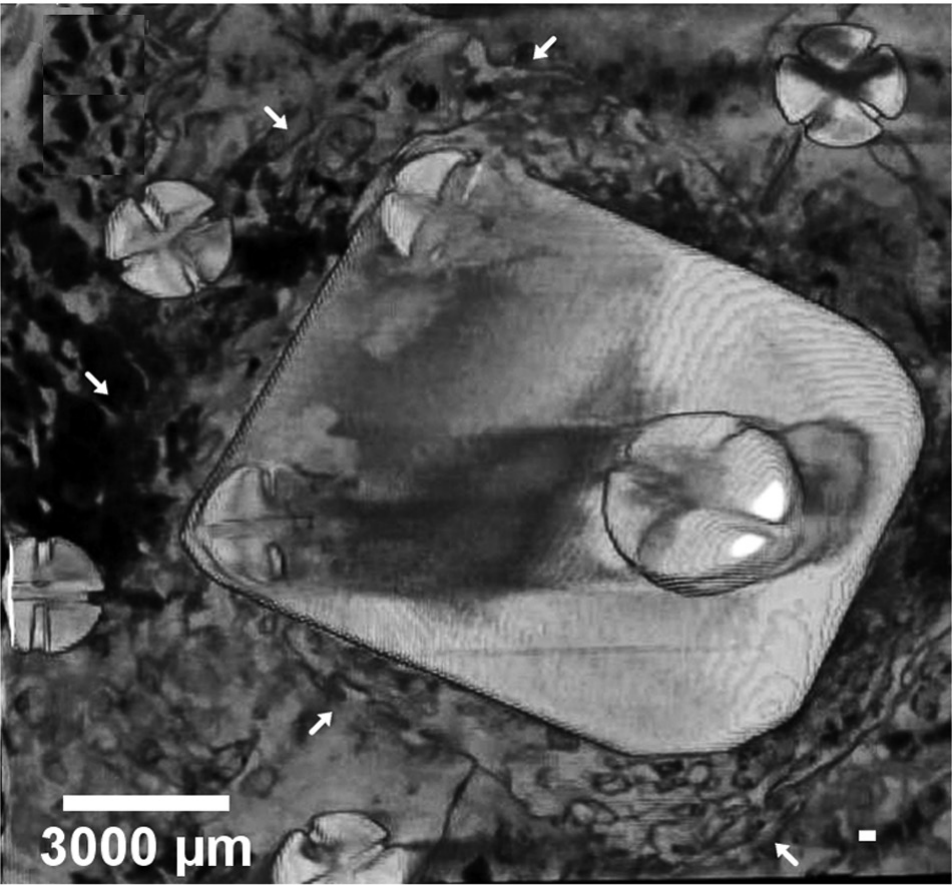
Top view shows the image of rectangular titanium plate surrounded by fixation screws. The area between the screws and the plate is occupied by new bone. This area represents the part outside the confines of the plate, however, protected by the expanded GBR membrane (group 2). GPR: guided bone regeneration.

After killing the animal, the calvarial part was harvested and then scanned in micro-CT. Lateral (Figure 4), top (Figure 5), and cross-sectional (Figure 6) views of the calvaria are shown. All examined calvarial bone surface showed significant bone apposition; however, the 4-month group showed new bone formation of thickness and radiopacity comparable to that of original bone (Figures 4(b) and 5(b)).

### Histology

**Figure 7.**
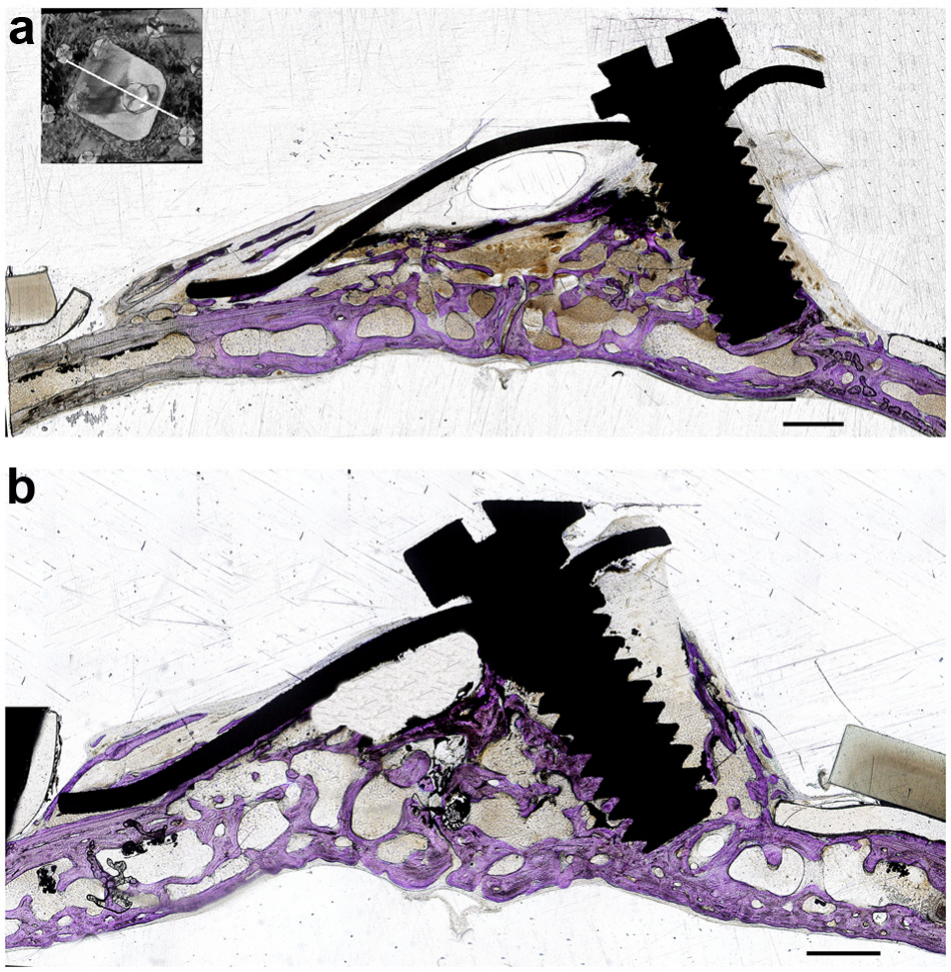
Histological longitudinal sections of the calvaria. Toluidine blue staining of the original bone and newly created space. In both groups, the new space is occupied by newly generated bone with an air space on the top. Note bone trabeculae creeping on the serrations of the elevation screw and along the inner surface of the silicone membrane: (a) group 1 and (b) group 2 scale bar =1.5mm.

**Figure 8.**
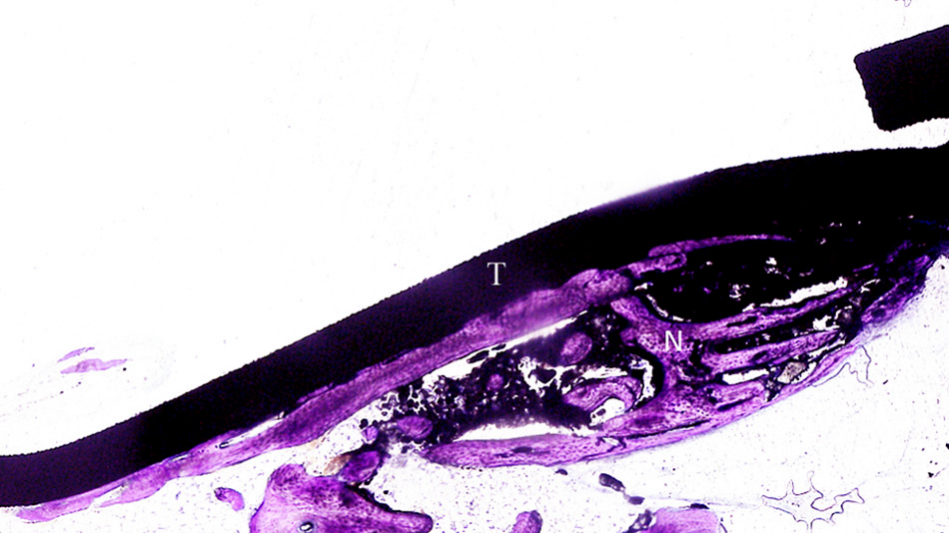
Longitudinal histological section of the distracted side. Toluidine blue staining section showing new bone trabeculae (N) proliferating on the inner side of the titanium plate (T) (group 2).

Microscopic examination showed that the newly generated bone tissue almost filled the space. Considerable amount of intramembranous bone trabeculae was formed under the titanium plate and silicone membrane. The interspace between trabeculae was filled with fat marrow and numerous blood vessels. Cortical bone plate was formed on the top of the newly generated bone tissue. Also, bone trabeculae were observed to creep over serrations of the titanium elevation screw (Figure 7(a) and (b)); bone trabeculae tend to proliferate along the inner wall of titanium plate (Figure 8) and silicone membrane. Calvarial bone showed mineralized bone with two thick compact layers surrounding marrow cavities. An empty space was always observed on the top of newly formed tissues (Figure 7(a) and (b)).

**Figure 9.**
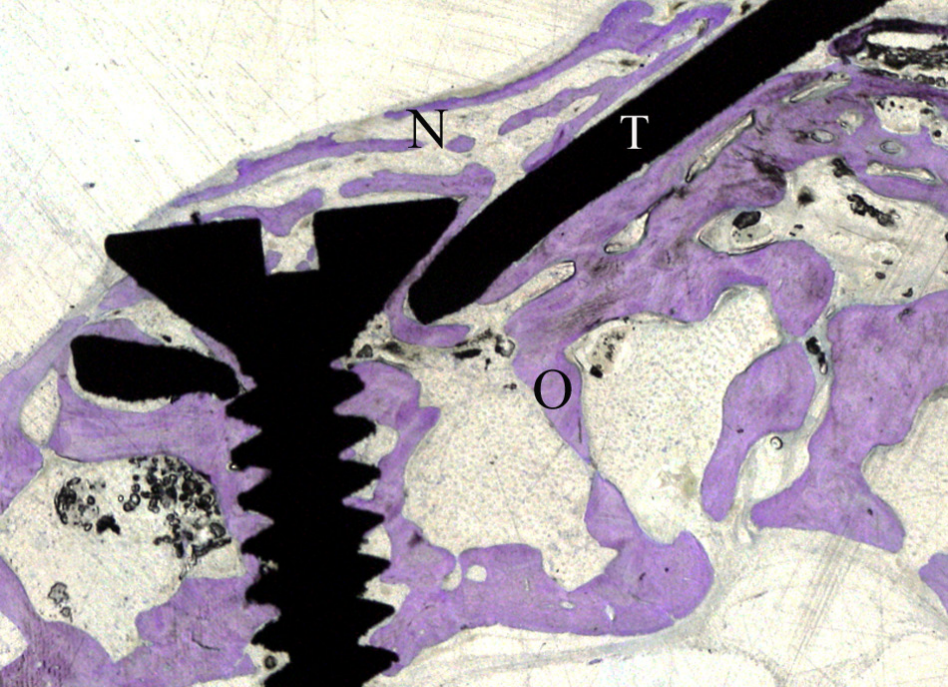
Longitudinal histological sections of the distracted side. Toluidine blue staining section showing bone trabeculae over the titanium plate. O: original bone; N: new bone; T: titanium plate.

**Figure 10.**
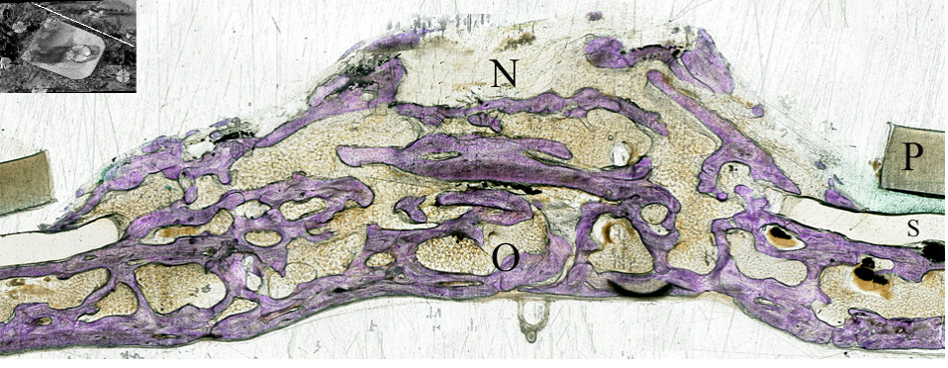
Longitudinal histological sections of the distracted side. Toluidine blue staining section showing new bone formed outside the titanium plate, however, protected by the silicone membrane (group 1). O: original bone; N: new bone; S: silicone ring; P: plastic ring.

**Figure 11.**
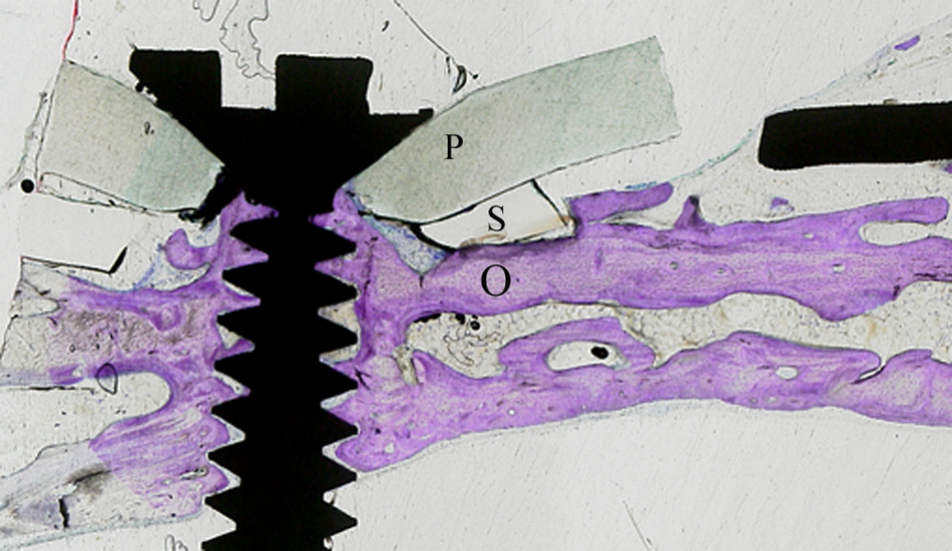
Longitudinal histological sections of the distracted side. Toluidine blue staining section showing the plastic ring pressing against the silicone membrane creating no gap between bone and original bone (group 1). O: original bone; S: silicone ring; P: plastic ring.

The generated new bone was also observed in the space outside the confines of the titanium plate, however, protected by the silicone membrane. Bone trabeculae over the plate at the anchored end (Figure 9) and in the peripheral areas of the device covered only by the silicone membrane (Figure 10) were observed. The plastic ring was always tightening the underneath silicone ring against the original bone creating no gap between the device and the calvaria (Figure 11).

It was remarkably noticed that bone trabeculae showed an increase in thickness while the intervening vascular connective tissue showed gradual decrease from 2- up to 4-month group (Figure 4(a) and (b)).

#### Micro-CT image analysis

The longitudinal section of the newly created space showed an unsymmetrical triangular shape in which slightly more than one-third of the newly created space was filled with newly formed bone tissue (32.9.4% in group 1 and 34.8% in group 2) (Figure 7(a) and (b)). However the peripheral one-third of the space showed nearly a segment of circle that is completely filled with new bone (Figure 10).

**Figure 12.**
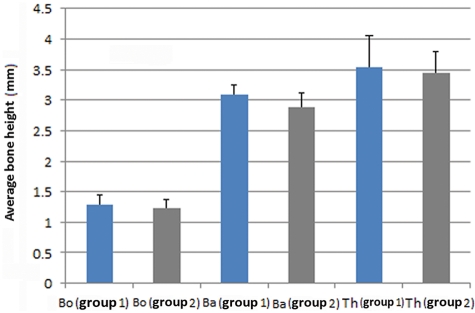
Comparison between the two groups regarding the original bone thickness of calvarium (Bo), calvarium bone thickness after augmentation (Ba) and maximum elevation height attained by the elevation plate (Th)

**Figure 13.**
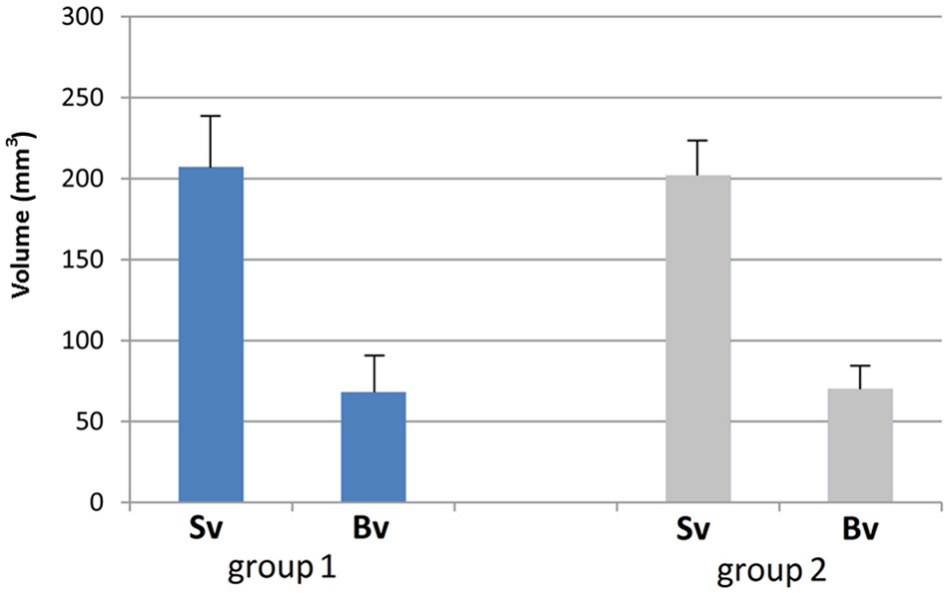
Comparison between space volumes attained (Sv) and new bone volumes gained (Bv) in( group I) and (group II)

Although a 5-mm-length screw was used for device activation, however, average height attained by the titanium plate was 3.45 mm in group 1 and 3.53 in group 2, as it was inserted in an inclined position. This elevation resulted into an increase in the original thickness of bone by 2.21 times in group 1 and 2.34 times in group 2 (Figure 12). Microradiographical calculation (3D image analysis) showed that considerable amount of de novo bone has been created (68.2 ± 22 mm^3^ in group 1 and 70.3 ± 14 mm^3^ in group 2) in the sizeable created spaces (207.1 ± 31 mm^3^ in group 1 and 202 ± 21 mm^3^ in group 2) (Figure 13). Statistical data were normally distributed; however, there was no significant difference between the two groups regarding the volume of the new bone (Vb) or the average height of augmented bone (Ba).

## Discussion

In this study, de novo bone formation beyond the genetically determined skeletal envelop has been achieved using the novel EGBR technique in a calvarial rabbit model. The bone was formed in a new in situ incubator model without ex vivo procedures or introduction of exogenous growth factors.

Upon activation of bone surface, biological healing process taking place on the activated surface is kept confined to the surface during the first week. After that the elevation plate is set to move upward, the membrane is gradually elevated and the space attains its maximum size in 5 days. Commonly, a scaffold introduced in site is responsible for maintaining the shape of the defect and preventing distortion of the surrounding tissue.^29^ However, in this experiment, the membrane supported the soft tissue and was responsible for providing the space shape and supporting the overlying soft tissue, hence providing a chance for a biologic matrix to be formed in the newly created space. It is now known that a biologic matrix is the actual scaffold even if a bone substitute is used. This matrix is composed of exposed collagen and the cell adhesion molecules, fibrin and fibronectin, from plasma and vitronectin secreted by platelets.^30^ The scaffold surface provided the solid base for accumulation and interaction of precursor cells with growth factors derived from blood platelets and released bone cell matrix contents.^29^ Viable cell sources include the endosteum lining the inner cortex, the trabeculae, and the Haversian systems in addition to undifferentiated marrow cells that have access to blood circulation pericytes.^31^ This explanation may clarify the generation of de novo supra skeletal bone out of local healing process occurring at the bone surface in the secluded space after the first week.

Normally, an influencing mechanism of GBR membrane placement may be that stimulating growth factors are locally concentrated in the operative site at inductive doses, leading to de novo neo bone formation.^32^ However, in this experiment, the healing site was given a growing space after abatement of progenitor cells and growth factors .The elastic property of the silicone membrane enabled to respond flexibly to elevation forces exerted by the titanium membrane and start to grow a space by the beginning of the second week.

The construction of the device combined the occlusiveness of the GBR membrane against cellular invasion and the flexibility of the periosteal distraction in creating new space. This enabled taking the advantages of both techniques and mitigating their limitation.

The prefabricated dome shape for de novo neo osteogenesis has been used in previous experiments using different materials.^33–35^ In this experiment, a tent shape has been used; however, it was created gradually on the operative site. This enabled much more appropriate management of soft tissue.

Although one of the prerequisites of GBR experiments is the membrane rigidity,^36^ we used in our experiment a very thin silicone membrane (0.05 mm thick). However, it showed no collapse, rupture, or discontinuity, and this is attributed to positioning it unstressed over the titanium plate, which supports most of it. The ability to create and maintain a given space with desired geometry adjacent to the parent bone surface is a critical requirement for successful GBR therapy.^36^ In this experiment, these goals were achieved. Moreover, shape of the created space can be greatly modified according to anatomical site.

Some GBR studies reported complete fill of the space with generated tissue,^19^ while other studies reported partial fill.^37^ In the present study, we observed empty space on the top of generated bone tissue in both groups, and this can be attributed to the considerable size of the newly created space in both groups with a minimum of 173 mm^3^ and maximum of 240 mm^3^ (Figure 13). These recorded volumes may exceed those reported by previous GBR studies.^34,36,37^ Definitely the amount of mesenchymal stem cells and growth factors accumulated on the surface inside the newly created space were insufficient to fill in the space completely with newly generated bone. However, previously reported that filling the GBR device with venous blood can probably increase bone formation in the space.^20^

The slight membrane hole dilatation that happened in three animals affected the amount of new bone formed in these animals. Compared to the normal average of other animals in the same group, the decrease was approximately 12% in group 1 and 18% in group 2. This incident further confirms maintaining strict seclusion against soft tissue while new bone grows in the space.

This experiment reported no significant differences between the two groups regarding bone heights and volumes attained. This might be owed to disuse bone resorption in group 2; however, histological observations in this group showed relatively wider trabeculae and diminished inter trabecular spaces.

Previous GBR experiments used tibia, ramus, and calvaria,^12,17–39^ while in this experiment, the posterior part of the calvaria has been used as operative site for its wide area and being less prone to infection. However, the calvarias lack mechanical stress that the newly formed bone needs to receive for maturation.

This experiment is consistent with Stevens et al.’s^3^ experiment, in which a space adjacent to bone surface is created by elevating the periosteum to be used as a bone bioreactor Stevens et al.’s Ref No 10. Also, it is in accordance with Simon et al.’s,^9^ experiment in which an in situ bioreactor is created by mechanical stimulation of cells. However, this experiment diverged from those two experiments in which it coaxed on the total exclusion of periosteum in creating the in situ bioreactor. All these mentioned experiments are in line with the principles of the tissue engineering approach “facilitated endogenous repair,” suggested by Evans et al.^8^ in terms of expedite, limited procedures, and refraining from vitro production of engineered tissue Evans et al Ref No 7. Moreover, in this study, procedures did not involve introduction of any genes, growth factors, or progenitor cells.

We believe that this technique may be appropriate to use in clinical and research fields. Employing this technique clinically to treat alveolar bone deficiency in vertical and horizontal aspects may sound applicable. It may offer an affordable, unsophisticated, and safe treatment option for unwealthy patient section. Furthermore, this technique may renovate the GBR technique from an exclusive dental treatment modality into an approach widely used in more diverse clinical fields. Our preliminary findings may strengthen the possibility for using this in vivo bone bioreactor in humans as a resource of easily harvestable engineered autogenous bone for autografting with little morbidity. Also, using as a bioreactor in animal models may give prospects to improve the quality of the engineered bone tissue. The penetrable nature of the secluding membrane provides the option of introduction of growth factors and stem cells. This can present a platform to further study the effect of these materials during different osteogenesis stages.

Simplifying this device may accelerate its use clinically. The polyethylene ring can be discarded and consequently the fixation mini screws. Their occlusive action can be compensated with a surplus of membrane that extends few millimeters beyond the site borders to protect it against soft tissue invasion. Further simplifying could include a biodegradable elevating plate.

The most important limitation of this study is that the number of rabbits used in each group was too small to clarify the changes according to the time period. There was no control group to compare the results; however, in our previous studies,^11,40^ we used devices to elevate the periosteum and overlying soft tissue without any secluding membrane in the same calvarial rabbit model.

In conclusion, this experiment reports creation of a sizeable secluded supraosteal regenerative space with high ability to control its shape. In future studies, we recommend perforation of the cortex or injecting aspirated peripheral blood to ensure complete fill of the space with blood and remove air.
